# Utilizing Extracellular Vesicles for Eliminating ‘Unwanted Molecules’: Harnessing Nature’s Structures in Modern Therapeutic Strategies

**DOI:** 10.3390/molecules29050948

**Published:** 2024-02-21

**Authors:** Monika Kisielewska, Katarzyna Rakoczy, Izabela Skowron, Julia Górczyńska, Julia Kacer, Agata Bocheńska, Anna Choromańska

**Affiliations:** 1 Faculty of Medicine, Wroclaw Medical University, 50-367 Wroclaw, Poland; monika.kisielewska@student.umw.edu.pl (M.K.); katarzyna.rakoczy@student.umw.edu.pl (K.R.); izabela.skowron@student.umw.edu.pl (I.S.); julia.gorczynska@student.umw.edu.pl (J.G.); julia.kacer@student.umw.edu.pl (J.K.); agata.bochenska@student.umw.edu.pl (A.B.); 2 Department of Molecular and Cellular Biology, Faculty of Pharmacy, Wroclaw Medical University, Borowska 211A, 50-556 Wroclaw, Poland

**Keywords:** extracellular vesicles, oxidative stress, endoplasmic reticulum stress, autophagy, mitochondrial-derived vesicles, aging process, age-related diseases

## Abstract

Extracellular vesicles (EVs) are small phospholipid bilayer-bond structures released by diverse cell types into the extracellular environment, maintaining homeostasis of the cell by balancing cellular stress. This article provides a comprehensive overview of extracellular vesicles, their heterogeneity, and diversified roles in cellular processes, emphasizing their importance in the elimination of unwanted molecules. They play a role in regulating oxidative stress, particularly by discarding oxidized toxic molecules. Furthermore, endoplasmic reticulum stress induces the release of EVs, contributing to distinct results, including autophagy or ER stress transmission to following cells. ER stress-induced autophagy is a part of unfolded protein response (UPR) and protects cells from ER stress-related apoptosis. Mitochondrial-derived vesicles (MDVs) also play a role in maintaining homeostasis, as they carry damaged mitochondrial components, thereby preventing inflammation. Moreover, EVs partake in regulating aging-related processes, and therefore they can potentially play a crucial role in anti-aging therapies, including the treatment of age-related diseases such as Alzheimer’s disease or cardiovascular conditions. Overall, the purpose of this article is to provide a better understanding of EVs as significant mediators in both physiological and pathological processes, and to shed light on their potential for therapeutic interventions targeting EV-mediated pathways in various pathological conditions, with an emphasis on age-related diseases.

## 1. Introduction

**Figure 1:**
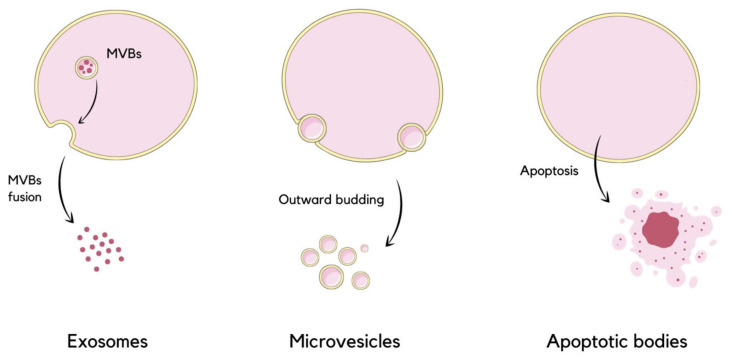
Schematic visualization of extracellular vesicles division based on their size and mode of release. Exosomes are released due to MVBs’ fusion with the plasma membrane, microvesicles are formed by the direct outward budding of the membrane, and the formation of apoptotic bodies occurs during apoptosis [4].

Extracellular vesicles (EVs) are nano-sized phospholipid bilayer-bond particles which are ubiquitous as they are released by almost all types of cells of multicellular organisms, enabling the process of discarding unwanted molecules and mediating intercellular communication. They are encountered in the integration of physiological homeostasis, as well as in pathological processes [1]. They form a highly heterogeneous group in terms of their size, cargo, membrane surface composition, biogenesis, and function [2,3]. Commonly, EVs are separated into subpopulations based on their size, which range from 30 to 1000 nanometers in diameter, or on the biogenesis and release mechanisms [1]. EVs are distinguished into three major subtypes, which are exosomes, microvesicles (MVs, shedding vesicles), and apoptotic bodies (ABs) (Figure 1). Exosomes are the smallest, with a diameter ranging from 30 to 150 nanometers. These particles are formed initially as intraluminal vesicles (ILVs) within multivesicular bodies (MVBs) invaginated from the endosomal membrane into the intraluminal space of the endosome. ILVs can be degraded in lysosomes or released into the extracellular matrix as exosomes upon MVBs’ fusion with the plasma membrane [4,5,6]. Microvesicles are between 100 and 1000 nanometers in diameter. These vesicles are secreted directly into extracellular space by the outward budding of the plasma membrane [2,4,7]. Apoptotic bodies are formed during programmed cell death by cell fragmentation. Recently, large oncosomes (LOs) have been included into EVs classification as the fourth subtype [4]. Oncosomes may be used as a term of MVs released from cancer cells, directly budding from their plasma membrane [8]. One study has proposed extended classification, which includes additional EV types such as autophagic EVs, matrix vesicles, and stressed EVs [9].

Various cell-specific molecules, such as proteins, lipids, and nucleic acids are incorporated into EVs during their biogenesis, predominantly selectively. The content of EVs is indicative of their heterogeneity, even among vesicles originated from the same cell type. Although the quantity of EV cargo is relatively small, the diversification and combination of specific molecules facilitate the recognition of EVs by target cells. Proteins accumulated within EVs vary among different types of vesicles. Commonly found proteins are responsible for biogenesis, EV formation, and release. Moreover, EVs entrap diverse types of tetraspanins (CD63, CD81, CD9), proteins associated with signal transduction (EGFR) and antigen presentation (MHC I, MHC II). The lipid composition of EVs often reflects the donor cell contents. However, particular lipids can be assembled within specific EVs, as vesicles formed of MVBs imply more phosphatidylserine, compared to the cellular plasma membrane, for their incorporation into target cell. Furthermore, lysobisphosphatidic acid is found on the exosome surface, in comparison to other cellular membranes. Genetic material enriched in EVs consists of various nucleic acids, primarily different types of RNAs, whose encapsulation is assumed to play a significant role in protecting EVs from degradation by RNase within extracellular space [1,3,7,10,11,12]. Contrarily, the content of apoptotic bodies differs from others vesicles, as they form during apoptosis. They are loaded with numerous cellular components, including micronuclei, chromatin fragments, cytosol, degraded proteins, DNA fragments, as well as organelles such as mitochondria or endoplasmic reticulum. Additionally, the ABs’ membrane differs from other cell membranes due to the lipid membrane rearrangement which occurs during apoptosis, as the phosphatidylserine is translocated from the inner to the outer layer, enabling the recognition of ABs by surrounding phagocytes [13,14].

Once the vesicles are released into the extracellular matrix (ECM), they can have an impact on the cells in the vicinity of the donor cell, or they can be transferred via circulation to distant cells. Moreover, a small amount of EVs dissolve in the ECM [15,16]. Vesicles can be internalized by target cells via multiple uptake mechanisms, including plasma membrane-EV fusion and various endocytosis processes, such as clathrin and caveolin-mediated endocytosis, lipid raft-mediated endocytosis, micropinocytosis, and phagocytosis, depending on the cell type [1,2,17]. The uptake is possible subsequent to the recognition of the vesicle. The interaction between the EV and the target cell occurs due to the presentation of specific molecules at both the EV and recipient cell surfaces [11]. As previously stated, the exposition of phosphatidylserine (PS) on the outer membrane of ABs leads to their phagocytosis, but interestingly, PS can be also exposed on microvesicles and exosomes originating from tumor cells [13]. The effect on the target cell may be conducted with a direct ligand-receptor binding process, leading to the stimulation of surface receptors, or by releasing EVs’ cargo into the cytosol of recipient cells. Molecules derived from EVs may trigger diversified intracellular responses, or they can cooperate in the formation of another EVs, which is defined as the recycling of intercellular communications [16,18,19]. Additionally, the surface of exosomes contains matrix-remodeling enzymes, which have an impact on both physiological and pathological conditions [20].

The role of extracellular vesicles has been widely examined by researchers. Besides their herein-discussed role of discarding unwanted molecules from cells, recent studies indicate that their potential is more comprehensive than previously assumed. EVs are important mediators in intercellular communication, both in physiology and pathophysiology, as they transfer their cargo from one cell to another. One way of unwanted molecules’ disposal is aforementioned phagocytosis of ABs, which consequently elicit selective elimination of cellular debris. However, that is not the solitary role of ABs. For instance, osteocytes-derived apoptotic bodies have been shown to trigger de novo bone resorption, whereas phagocytosis of ABs released from hepatic stellate cells (HSC) leads to HSC survival [2]. The ILVs generation also does not always lead to exosomes release, as ILVs can undergo retrofusion with the MVBs’ membrane and, in the aftermath, MVBs may fusion with lysosomes, inducing their cargo degradation [5]. A separate mechanism of undesirable particles removal consists of the degradation of mitochondrial-derived vesicles (MDVs), which are packed with oxidative proteins and lipids, by lysosomes and peroxisomes [21]. The intercellular communication pursued by EVs may have both positive and negative after-effects, depending on various factors. Extracellular vesicles have an essential impact on plentiful physiological processes, such as embryonic growth, pregnancy, hemostasis, and metabolism. EVs may also contribute to the pathogenesis of various diseases, including cancer, Alzheimer’s disease, hypertension, and diabetes [20]. Furthermore, exosomes have the capacity to alter the immune reaction [2,22].

## 2. EVs as Integrators of Homeostasis

### 2.1. EVs under Oxidative Stress Conditions

Oxidative stress is a physiological anomaly that occurs as a consequence of imbalance between oxidants and antioxidants in a biological system [23]. Exacerbation of oxidative stress leads to various diseases, disorders, and aging [24]. It is associated with nearly all diseases, including cancer [25,26]. A recent study concluded that 50% of the circa 200 drugs approved by the Food and Drug Administration to treat cancer can generate oxidative stress. There is higher possibility that complications from cancer treatment will occur while cancer patient respond correctly to the therapy. Complications can moderately appear from oxidative stress-induced noncancerous tissue damage, diminishing the quality of life of cancer survivors [27]. Oxidants can lead to cardiotoxicity, a major cause of death in cancer survivors. That is why early cardioprotective intervention is a goal. The research has indicated that EVs carry out the removal of oxidized proteins during doxorubicin (DOX)-induced cardiotoxicity. Biomarkers different from EVs for cardiac injury are detectable only after tissue injury has occurred [28].

**Figure 2:**
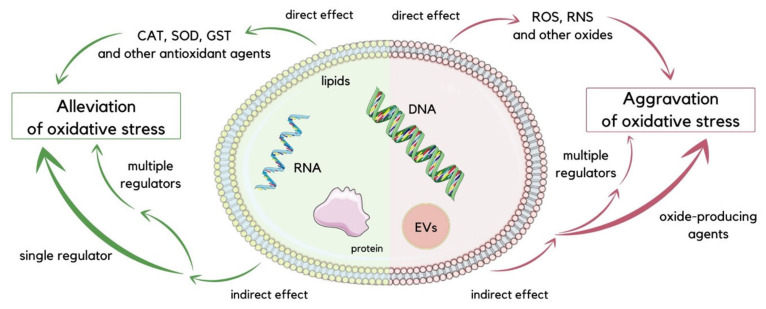
Schematic visualization of direct and indirect ways of oxidative stress regulation executed by EVs. To reduce oxidative stress, EVs can directly deliver antioxidant compounds such as CAT, SOD, and GST, or indirectly transport single or multiple regulators to target cells. Conversely, for the aggravation of oxidative stress, EVs can deliver oxides such as ROS and RNS directly to recipient cells, while oxide-producing agents or multiple regulators can be delivered indirectly [25].

As evidenced by recent studies, EVs regulate oxidative stress conditions [29,30,31,32]. It has been suggested that cells take part in removing oxidized toxic molecules using EVs. Cellular oxidative stress leads to the formation of modified molecules such as oxidized lipids, oxidized proteins, and mtDNA fragments, which are sorted into EVs for removal. These molecules can reflect on the origin and oxidative status of releasing cells [33]. Numerous studies have speculated on the role of EVs in redox signaling and oxidative-stress-related pathologies [30]. EVs balance oxidative stress in direct and indirect ways. Directly, they may provide antioxidants or oxides to the recipient cell, mitigating or exacerbating oxidative stress. EVs deliver oxides, such as reactive oxygen species (ROS) and reactive nitrogen species (RNS), or antioxidant molecules such as superoxide dismutase (SOD), catalase (CAT), glutathione S-transferase (GST), glutathione peroxidase (GPX), peroxiredoxin (PRDX), and thioredoxin (TRX) [34]. These antioxidant enzymes contained in EVs can relieve oxidative stress under diversified pathological conditions (Figure 2). For instance, T lymphocytes release EVs that can distribute antioxidant enzymes, such as SOD isoforms and CAT, to human umbilical vein endothelial cells (HUVECs) and scavenge ROS [35]. Stem cells have been shown to be one of the main sources of antioxidant enzyme-loaded EVs. Studies suggested that EVs which contain manganese-SOD (MN-SOD) secreted by human mesenchymal stem cells (hMSCs) can reduce oxidative stress in hepatic ischemia-reperfusion injury [36]. Mediation of CAT is accomplished with EVs secreted from hMSCs, which can protect hippocampal neurons from oxidative stress [37]. Endothelial EVs bring functional endothelial nitric oxide synthase (eNOS) to balance the eNOS/Akt signaling pathway, securing endothelial cells from oxidative stress [38]. Indirectly, EVs work through delivering factors that regulate oxidative-stress-related pathways to the recipient cells. They can distribute drugs to target cells and control signaling pathways associated with oxidative stress. EVs secreted by hMSCs can mediate the nuclear factor erythroid 2-related factor (Nrf2) signaling pathway to reduce ROS generation in H_2_O_2_-stimulated keratinocytes or UV-irradiated mice skin [39]. The p38MAPK pathway may also be suppressed by increasing GSH levels with EVs [40]. Elimination of ROS and reduced activity of myeloperoxidase (MPO) are possible because of EVs derived from human placental mesenchymal stem cells (hPMSCs), which productively suppress oxidative stress [41]. A study shows that blood-derived EVs from healthy individual possess neuroprotective effects by regulating oxidative stress in a mice model of Parkinson’s disease [42]. Oxidative stress injury of intestinal stem cells’ (ISCs) mediation via the Wnt/β-catenin signaling pathway is reduced by human-milk-derived EVs [43]. Unfortunately, there are various types of EVs, and some of them may complicate the regulation of oxidative stress [25]. EVs may also intensify the oxidative stress. It is rarely reported that ROS-loaded Evs result in a direct effect on recipient cells because of the short life span of ROS. Indirectly, EVs may aggravate oxidative stress by producing ROS using the loaded enzymes. NADPH oxidases (NOX) subunits delivered by endothelial EVs produce ROS to engage in endothelial damage [44]. Human umbilical vein endothelial cells (HUVECs) treated by hypoxia/reoxygenation release EVs that were used in the delivery of ROS into H9C2 cardiomyocytes, resulting in ROS overload and consecutive oxidative stress [45]. The multitarget control ability of EVs loaded with gene drugs is observed, and it may induce numerous target gens to implicate oxidative stress. For instance, microRNA-34c can be delivered from ionizing irradiated mouse embryo fibroblasts (MEFs) with EVs into unirradiated cells, possibly triggering a cascade of gene expression shift, resulting in an ROS increase and inducing oxidative stress [46]. EVs secreted by ketamine-injured human uroepithelial cells (SV-HUC-1) contain specific miRNAs, which enhance oxidative stress by mediating the P38/NFκB pathway [47]. It is reported that platelet-derived EVs can form ROS by the mediation of NOX and, as a consequence, induce vascular cell apoptosis in severe sepsis patients [48]. Previous research also has shown that EVs derived from septic shock patients present protective effects on vascular function [49]. In addition, immune cells can be stimulated by EVs released under oxidative stress conditions. Human embryonic kidney cells (HEK293 cells) that are treated with Ca^2+^ ionophore or synthetic EVs with 16-lipoxygenase generate EVs with lipid peroxidation products on their surfaces. These stress-mediated EVs stimulate Toll-like receptor 4 (TLR4), activating nuclear factor kappa-light-chain-enhancer of activated B cells (NFκB), which results in pro-inflammatory cytokine release from macrophages [50].

Furthermore, EVs mediate intercellular communication, which is crucial in the progression of many oxidative stress-related diseases, including cancer-microenvironment crosstalk [30,51]. Neighboring cells and their regional microenvironment actively communicate with tumor cells during primary tumor formation. It is suggested that EVs participate in multiple steps during the invasive process, and conceivably enhance early steps involved in metastasis [51]. Cancer cells take advantage of EVs to remove intracellular chemotherapeutic drugs and support chemo-resistant phenotypes. Previous studies have shown that human pancreatic cancer cells release MVs, leading to the removal of gemcitabine (GEM), a chemotherapeutic that inhibits DNA synthesis. The level of drug resistance and the inhibition of MV release sensitize cells to GEM [52].

**Figure 3:**
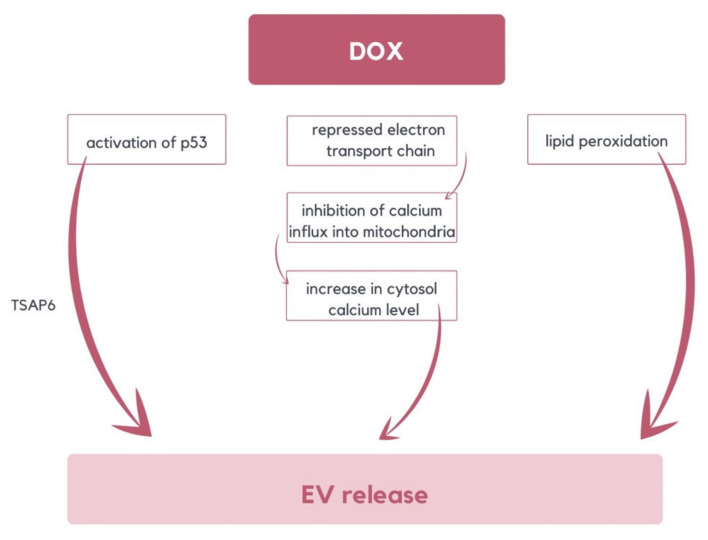
The diagram illustrates various mechanisms of DOX-induced EVs release. These mechanisms include the activation of TSAP6 by p53, lipid peroxidation, and an increase of cytosolic calcium level resulting from the repression of the electron transport chain [33,53,54,55,56].

To maintain cellular homeostasis, the cell can remove oxidized proteins by releasing EVs, which can be transferred to neighboring or distant cells, thereby instigating an intercellular oxidative stress response. During DOX-induced oxidative stress, EVs constitute an alternative pathway for toxic proteins’ removal in cells. DOX can promote EV release in diverse ways. MVBs production can be increased via tumor suppressor-activated pathway 6 (TSAP6) activation, stimulated by DOX activation of p53 [53,54]. DOX interferes with the electron transport chain, leading to an inability to maintain the mitochondrial membrane potential and, consequently, the inhibition of Ca^2+^ influx into mitochondria [55]. It results in an elevation of cytosolic Ca^2+^, a crucial event that induces MV release. Moreover, DOX-induced lipid peroxidation may influence membrane curvature and promote EV formation (Figure 3) [56]. The mechanism of DOX-promoting EVs is based on oxidative stress. Therefore, this indicated concept can be applied to alternative chemotherapeutic substances that generate ROS/RNS as well [33].

Moreover, EVs can potentially be a better biomarker for oxidative stress than conventional serum biomarkers. Their main pros include stability and distribution in many body fluids such as blood, urine, saliva, milk, lymph, ascites, and amniotic fluids. Additionally, EVs are protected from enzymatic degradation, and messages can be delivered with high specificity to recipient cells via receptor-mediated endocytosis [57]. EVs are highly associated with tissue redox status, as oxidized molecules are sorted within cells into EVs for removal. EVs containing brain/heart glycogen phosphorylase (GP) were successfully established as early and sensitive biomarkers of cardiac injury [28,33].

### 2.2. Interactions between Endoplasmic Reticulum Stress and EVs

The endoplasmic reticulum (ER) constitutes a dynamic membranous labyrinth consisting of branching tubules and placoid sacs. Among its numerous functions, the most distinctive one is post-translational protein modification, which includes specific folding and oligomerization, and thus plays a significant role in sustaining homeostasis [58]. As ER capacity is limited, an excessive influx of burgeoning proteins is able to create an imbalance that causes misfolded proteins to overly accumulate in the lumen of the organelle. This situation results in a phenomenon defined as ER stress, which might lead to either the induction of apoptosis or adaptation for survival [59]. The second scenario is possible if transcriptional and translational cell programs are regulated in a way that allows them to manage the folding defect.

**Figure 4:**
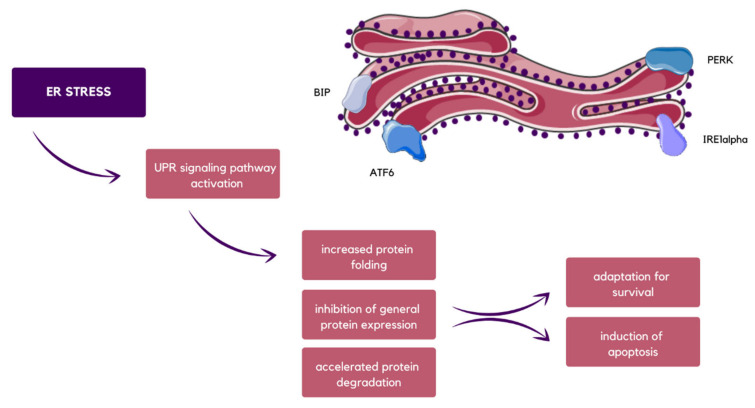
Schematic visualization of ER stress possible results, which in the end leads either to adaptation for survival or induction of apoptosis, as well as the proteins that are directly involved in the UPR signaling pathway, which are BIP, ATF6, PERK, and IRE1α [59,62,64,65].

Under homeostatic conditions, glucose-regulated protein 78 (GRP78) is associated with inositol-requiring 1α (IRE1α), activating transcription factor 6 (ATF6) and pancreatic endoplasmic reticulum kinase (PERK), which are transmembrane mediators. Under stress, GRP78 dissociates, and therefore the unfolded protein response (UPR) signaling pathway is activated. Then, IRE1α undergoes an autophosphorylation process, causing X-box binding protein 1 (XBP1) frameshift and translation of its isoform with transcription factor activity [60,61]. Simultaneously, the release of binding immunoglobulin protein (BIP) precedes ATF6 cleavage with Golgi apparatus proteases. In the divided form, ATF6 cooperates with active XBP1 to trigger ER chaperones and enzymes encoding gene transcription, which supports protein folding as well as their secretion or degradation [62]. What is more, after BIP release, eukaryotic initiation factor 2α (eIF2α) is phosphorylated by PERK, and in this way, general protein expression is suppressed, preventing excessive inflow of proteins into the saturated ER, and thus promoting cell survival (Figure 4). Nevertheless, if prolongedly uncompensated or very intensified, ER stress might lead to UPR signaling pathway failure, which consequences in cell death [63,64,65].

Imbalances in ER function underlie the pathogenesis of a broad spectrum of diseases. Serving as a protein-folding center and a dynamic storage of intracellular calcium supplies, the ER is significant in the development of autoimmune diseases, cardiovascular diseases, metabolic disorders, and cancer [66,67,68,69,70]. Extracellular vesicles, which transmit various molecules such as lipids, proteins, and nucleic acids amongst cells, play an important role in enumerated conditions. Importantly, the formation of EVs depends on ER function. ER stress promotes the transmission of pathological stimuli to EVs, which are then distributed to their target cells, thereby contributing to disease development. Furthermore, EVs can transmit pathological stimuli to healthy cells, promoting ER stress. Therefore, ER stress simultaneously constitutes a possible cause and consequence of particular diseases, while EVs cause ER stress as well as constituting its derivatives.

**Figure 5:**
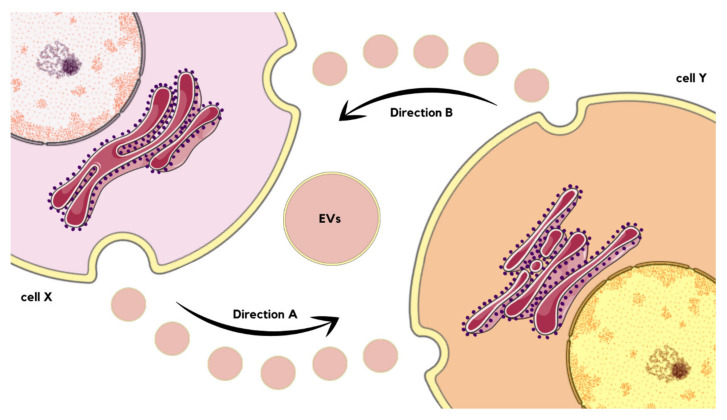
Schematic visualization of interactions between EVs’ release and ER stress occurrence, as well as transmission. If the cell X releases EVs that are internalized by cell Y (Direction A), there is a possibility that the content of EVs might cause ER stress in the cell Y. Under stress, cell Y releases EVs internalized by cell X (Direction B), which might transmit ER stress to cell X or cause another effect [70,73,74].

In one study, EVs generated from cultured endothelial cells subjected to thapsigargin-induced ER stress were observed to trigger ER stress in endothelial cells, suggesting a two-directional relationship between them. Moreover, ER-stress-generated EVs were proven to impair the angiogenic capacity of human umbilical vascular endothelial cells (HUVECs) independently from cell survival or autophagy mechanisms [71]. If the cell X releases EVs that are internalized by cell Y (direction A), there is a possibility that the content of EVs might cause ER stress in the cell Y. Of course, a wide range of different outcomes is also probable, such as autophagy, angiogenesis, or immune response. Nevertheless, under stress, cell Y releases EVs internalized by cell X (direction B), which might transmit ER stress to cell X as well as result in other consequences (Figure 5) [72,73,74]. As can be seen, the interaction between ER stress and EVs are not unidirectional, but rather forms a complex bilateral cyclic process that needs to be further investigated and understood. Future research might target particular parts of the cycle as a novel therapeutic approach, as the network of dependencies between discussed phenomena underlays pathogenesis of numerous diseases, examples of which are described below [75].

Cancer EVs are able to induce tumorigenesis through multiple pathways, involving the transfer of miRNAs, which can either regulate gene expression of the recipient cell [76,77], be translated into functional proteins [78,79], or active proteins influencing cell physiology in a direct way, contributing to tumor growth and immune response suppression [78,80]. The durability of changes caused by EV-mediated horizontal transfer of oncogenic molecules has long been questioned, as the described molecules tend to trigger alterations that fade when EV influence declines [81]. However, evidence of their capability to trigger lasting changes has been provided. In one study on bladder cancer, prolonged exposure to cancer EVs was observed to result in one of UPR pathways, pro-survival IRE1α pathway induction, inflammatory cytokines release, DNA damage response (DDR) activation, and ultimately malignant transformation [82]. The EVs-inducted UPR pathway might, therefore, be considered a novel tumorigenesis mechanism founded upon EVs–ER stress correlation.

In the pathogenesis of endothelial dysfunction, the dependencies between EVs and ER stress are of great importance. ER-stress-dependent EVs from smooth muscles and human aortic endothelial cells (HAoECs), induced by mechanical stretch, have been isolated. Chemical chaperone 4-phenyl butyric acid (PBA) is able to prevent ER stress. When applied before inducing ER stress through mechanical stretch, it was found to decrease both EVs’ release and pathological outcomes mediated by extracellular vesicles [83]. Another study has demonstrated the ER stress involvement in endothelial dysfunction caused by EVs derived from metabolic syndrome patients and apoptotic lymphocytes. During the research, in vitro treatment of HAoECs with EVs resulted in the activation of all UPR pathways, involving PERK, IRE1α, and ATF6. Moreover, reduced NO bioavailability, as well as impaired endothelium-dependent vasodilatation in vivo in mice, has been noticed. Importantly, tauroursodeoxycholic acid (TUDCA), an ER stress inhibitor, reversed all observed changes [84].

Among cardiovascular conditions, the one significantly connected with EVs–ER stress correlation is vascular calcification (VC). VC describes the process of calcium phosphate crystals’ deposition in the extracellular matrix of the blood vessels wall, mediated by vascular smooth muscle cells (VSMCs). VC increases cardiovascular risk, as well as constituting a separate risk factor of cardiac events [85,86,87]. In one of the research, ER stress markers, PERK, ATF4, ATF6, and GRP78 mRNA expression levels have been measured in both healthy and calcified vessels. Compared to healthy vessels, calcified ones presented decreased levels of PERK and increased deposition of GRP78 in the matrix [66,88]. Interestingly, siRNAGRP78 knock-down resulted in decreased calcification. ER stress induction turned out to aggravate VSMC-mediated calcification, as well as elevate the expression of osteogenic markers, such as osteoprotegerin (OPG) and alkaline phosphatase (ALP). Moreover, ER-stress-intensified EVs release via sphingomyelin phosphodiesterase 3 (SMPD3). In the end, EVs turned out to play crucial role in ER-stress-dependent propagation of the VC. ER-stress-induced release of GRP78-packed EVs via the PERK-ATF4 pathway directly contributes to calcification. The described model, interestingly, refers to a common anticoagulant, warfarin, which induces ER stress and aggravates VC via an increase in EVs release [66].

### 2.3. Interactions between Autophagy and Exosomes

**Figure 6:**
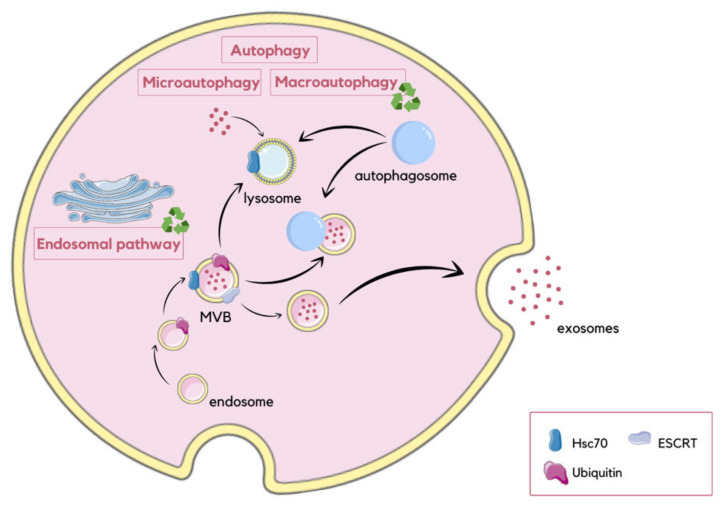
Schematic visualization of different autophagy pathways and their association with exosome release. During macroautophagy, entire regions of the cytosol are enclosed within autophagosomes, which subsequently fuse with MVBs or lysosomes, resulting in the degradation of their contents. In contrast, microautophagy involves the internalization of small cytoplasmic components through the inward invagination of the lysosomal membrane. CMA involves the degradation of unfolded proteins, and is mediated by the binding of hsc70 to the lysosomal membrane. Autophagy regulates exosome secretion through autophagy-related proteins located on the membrane of MVBs. In this process, the ESCRT machinery and hsc70 are involved, facilitating the incorporation of proteins into ILVs [91,95].

Autophagy is a degradative pathway in which the selective removal of damaged organelles improves their function. The process occurs constitutively, or is inducible by cellular stress incidents, to limitations of various types of nutrients like amino acids, growth factors, oxygen, and energy, as well as excessive ROS or DNA damage [89]. Degradation of self-components is a required survival response against starvation conditions, as it enables recycling of macromolecules to provide new nutrients and energy. Autophagy also eliminates potentially toxic aggregates. Moreover, it limits the accumulation of ubiquitinated proteins, which contributes to protein homeostasis. This degradative pathway is responsible for selective removal of dysfunctional mitochondria, which release proapoptotic factors and generate reactive oxygen species (ROS) [90,91]. In addition, it has been observed that autophagy is induced after ER stress as part of the previously described UPR pathway [92]. This type of autophagy protects cells from apoptosis related to ER stress. Currently, three autophagy pathways have been observed, which are macroautophagy, chaperone-mediated autophagy (CMA), and microautophagy. Macroautophagy occurs when entire cytosolic regions are sequestered in autophagosomes. These vesicles can then fuse with MVBs or lysosomes, providing hydrolytic enzymes that degrade the contents of autophagosomes [93]. CMA is a more selective pathway that does not require membrane reorganization. Chaperone hsc70 attaches to the lysosome-associated membrane protein 2A (LAMP-2A) immediately after recognition of cytosolic substrate proteins having KFERQ-like pentapeptide motifs [94]. Microautophagy involves the engulfment of small plasma cytoplasmic components by invagination of the lysosomal membrane. It has been shown that autophagic pathways are associated with exosomal pathways, as autophagy can contribute to exosome biogenesis. The endosomal sorting complex required for transport (ESCRT) enables the formation of early and late endosomes, and, consequently, the formation of MVBs. Moreover, ESCRT regulates the sorting of ubiquitinated proteins into the ILVs within MVBs. The process of selective encapsulation of proteins into exosomes during their formation has been suggested to be a form of endosomal microautophagy. In this type of microautophagy, proteins are transported into ILVs with the assistance of the ESCRT and chaperone hsc70. However, unlike classical CMA, hsc70 does not require the presence of LAMP-2A in order to bind to the endosomal membrane. In addition, autophagy-related proteins, such as autophagy related 5 (ATG5), autophagy related 16 like 1 (ATG16L1) and microtubule-associated protein 1-light chain 3 beta (LC3B), are located on the MVB membrane, and have been proposed to play a role in the exosome secretion [91,95] (Figure 6). Furthermore, exosomes may also have an impact on autophagy processes, as they release their contents into target cells. The released cargo, primarily miRNAs, can either upregulate or inhibit autophagy via mammalian target of rapamycin (mTOR) and Beclin-1 signaling pathways [95].

In healthy cells, the homeostatic activity maintained by autophagy creates a hefty barrier against malignant transformation. Properly, many oncoproteins inhibit, and several oncosuppressor proteins promote, autophagy. Furthermore, autophagic response is required for optimal anticancer immunosurveillance. On the other hand, autophagy is a way of dealing with intracellular environmental stress in neoplastic cells, thus favoring tumor progression. Therefore, it can be observed that in some cases, oncogenesis occurs with an interim inhibition of autophagy or enhancement of molecular functions that antagonize the oncosuppressive effect [96].

Phosphorylation of eukaryotic translation initiation factor 2α (eIF2α) at serine 51 plays a significant role in autophagy regulation. Cells that carry a nonphosphorylatable mutant of eIF2α (S51A) fail to promote autophagy in response to starvation. This phosphorylation integrates different types of environmental and endogenous stress signals above ER stress, such as amino acid deprivation, exposure to double-stranded viral RNA, osmotic stress, UV light exposure, heme deficiency, hypoxia, and oxidative stress [97,98]. These diverse signals activate four different eIF2α kinases, including PERK, general control nonderepressible-2 (GCN2), heme-regulated inhibitor (HRI), and protein kinase R (PKR). PERK is activated by ER stress, radiation, or hypoxia, GCN2 is activated by uncharged tRNAs in amino acid-starved cells, HRI by heme deficiency in erythroid precursor cells, and PKR by double-stranded RNA and, in some contexts, ER stress. Yeast GCN2, mammalian PKR, and PERK have been shown to be necessary for autophagy induced by starvation, viral infection, and ER stress, respectively. Therefore, eIF2α kinases are said to regulate autophagy both in the UPR and in other stress states [98].

### 2.4. Mitochondrial EVs and Inflammation

**Figure 7:**
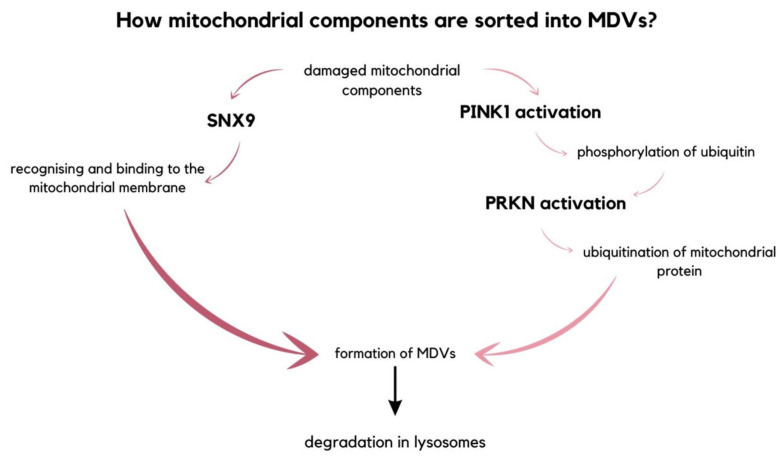
The diagram picturing two alternative pathways of sorting damaged mitochondrial components into mitochondrial-derived vesicles. Either is the MDVs formation initiated by SNX9 that recognizes and binds to the mitochondrial membrane, or the fragments intended for degradation are decomposed in lysosomes, which is regulated by PRKN [102,103,104].

Mitochondria are organelles enclosed by a double membrane and are thought to have originated from bacteria [99]. They play a vital role in numerous metabolic processes, including the production of ATP, biosynthetic intermediates, and apoptosis induction [100]. Additionally, mitochondria impact the maintenance of homeostasis by regulating communication between cells [101]. It is crucial to remove damaged cells to ensure a healthy population and proper function. For instance, impaired mtDNA, oxidized proteins, and lipids can disrupt mitochondrial functions. To eliminate and deliver these damaged molecules to lysosomes for degradation, mitochondria form MDVs [21]. If left unchecked, these impaired molecules can act as damage-associated molecular patterns (DAMPs) and lead to inflammation [102]. There are two alternative pathways through which mitochondrial components are sorted into MDVs. The first pathway involves an endocytic accessory protein, sorting nexin 9 (SNX9), recognizing and binding to the mitochondrial membrane to initiate the formation of MDVs. The second pathway involves the degradation of mitochondrial fragments directly in lysosomes, regulated by the mitophagy mediator Parkin (PRKN). Parkin is an E3 ubiquitin ligase that is recruited to the damaged mitochondria and mediates the ubiquitination of mitochondrial proteins. PRKN is activated by the phosphorylation of ubiquitin initiated by PTEN-induced kinase 1 (PINK1). PINK1 accumulates on the outer mitochondrial membrane due to its depolarization (Figure 7) [102,103,104].

Immune cells are equipped with a range of Pattern Recognition Receptors (PRRs), including Toll-like receptors (TLRs). These receptors have the ability to recognize mitochondrial DAMPs, such as oxidized cardiolipin, a phospholipid found in the inner membrane of mitochondria and mtDNA. During the course of certain diseases, these molecules are released into the bloodstream and activate the production of proinflammatory cytokines [105]. Additionally, mitochondria are lately considered to be the main source of intracellular DAMPs [106]. Under physiological conditions, DAMPs cannot bind to PRR-containing subcellular compartments [107]. During mitochondrial outer membrane permeabilization (MOMP), soluble molecules diffuse from the intermembrane space through the permeable outer membrane to the cytosol, thus activating apoptosis [108]. MOMP-induced inflammation is regulated by caspases. Inhibition of caspases leads to increased cytokine production and subsequent type I interferon response induced by apoptosis [109]. TLR9 is a receptor present in different immune cells that recognizes unmethylated CpG DNA, including mtDNA. The immune response is initiated when mitogen-activated protein kinase (MAPK) and NF-kB are activated due to the escape of mtDNA from autophagy [110,111]. PRRs have been identified in the cytoplasm, including nucleotide-binding oligomerization domain-like receptors (NLRs). Upon activation, certain NLRs are capable of assembling into multimeric complexes known as inflammasomes [112]. The inflammasome protein complex can activate caspase-1, which in turn triggers the secretion of pro-inflammatory cytokines, specifically IL-1β and IL-18 [110]. Studies have shown that, as people age past 50, their levels of circulating mtDNA molecules progressively increase and become associated with chronic low-grade inflammation [113]. The interaction between mitochondrial DNA and GMP-AMP synthase-stimulator of interferon genes (cGAS-STING) can also potentially induce an inflammatory response. The protein cyclic GMP-AMP synthase (cGAS) functions as a sensor of displaced DNA within the cytoplasm, binding to mitochondrial DNA to catalyze the formation of 2′3′ cyclic GMP-AMP (cGAMP) upon detecting such DNA misplacement. cGAMP, in turn, acts as a second messenger by binding to STING, ultimately facilitating the transcription of IFN stimulatory genes (ISGs) and type I interferon through the phosphorylation of TANK-binding kinase 1 (TBK1) and of interferon regulatory factor 3 (IRF3) [110,114]. The production of mitochondrial-derived vesicles is predominantly associated with elevated levels of intracellular reactive oxygen species, indicating their crucial role in the quality control mechanism that complements mitophagy. Mitochondrial reactive oxygen species (mtROS) play a crucial role in activating inflammasomes by acting as mitochondrial damage-associated molecular patterns (mtDAMPs). mtROS can cause oxidative damage to mitochondrial DNA, proteins, and membranes, which can change the structure and function of the organelle. This damage can lead to an increase in mtROS levels, particularly during mitochondrial malfunctioning. Elevated mtROS levels can activate redox-sensitive transcription factors, such as NF-kB, which may result in the production of pro-inflammatory cytokines [115,116,117].

### 2.5. Extracellular Vesicles as Ferroptosis, Pyroptosis, and Necroptosis Mediators

Regulated cell death (RCD) can be divided into non-apoptotic and apoptotic pathways. Examples of non-apoptotic pathways include ferroptosis, pyroptosis, and necroptosis [118]. Ferroptosis is caused by the accumulation of ROS and iron-dependent lipid peroxidation. The accumulation of Fe^2+^ can trigger the Fenton reaction, resulting in the overproduction of ROS, which then interact with polyunsaturated fatty acids (PUFAs) on the cell membrane. This process is characterized by disintegration of the plasma membrane, swelling of the cytoplasm and organelles, and condensation of chromatin. Moreover, it leads to significant alterations in mitochondrial morphology and an increase in the number of intracellular autophagosomes. Pyroptosis is initiated through the activation of diverse caspases, including inflammasome-mediated caspase-1, ultimately leading to cell death by membrane perforation. Disintegration of the membrane results in the release of cellular content, which eventuates in inflammation [119]. Necroptosis is activated by ligands of cell death receptors, such as tumor necrosis factor (TNF) and interferon (INF). It is mainly mediated by receptor-interacting serine/threonine kinase 1 (RIPK1), kinase 3 (RIPK3), and Mixed Lineage Kinase Domain-Like (MLKL). This process is marked by the swelling of the affected cell, rupture of the cell membrane, and outflow of cytoplasmic contents, resulting in inflammation and tissue damage [119,120].

Extracellular vesicles have been identified as potential mediators of non-apoptotic forms of regulated cell death by transferring a wide range of key regulators. Utilizing EVs to regulate non-apoptotic RCD offers a promising approach to treating human diseases. EV-mediated ferroptosis is a process that contributes to both tumor resistance and T-cell immunity. Certain compounds and medications, such as artesunate, erastin, lyrocrine, or altretamine, have been found to trigger ferroptosis in cancerous cells. Utilizing EVs to deliver these drugs and induce ferroptosis may potentially be a more efficient method than using free drugs alone. Expertly designed exosomes possess significant therapeutic potential across various types of cancer. Moreover, EV-mediated ferroptosis has been found to alter both chemoresistance and radioresistance in diverse tumors, such as lung and gastric cancers [121]. EV-mediated pyroptosis may have an impact on cancer progression by transporting molecules such as bioactive particles or drugs to target cells. For instance, cervical cancer may be treated by targeting nicotinamide adenine dinucleotide-dependent deacetylase (SIRT 1) due to its high expression. Elevated SIRT1 expression inhibits the AIM2 inflammasome, a mechanism that suppresses pyroptosis while promoting the proliferation of cancer cells. Providing EVs with AIM2 inflammasome proteins was found to induce pyroptosis in cancer cells by downregulating SIRT1 [121,122]. Necroptosis acts as an alternative form of programmed cell death that surpasses resistance to apoptosis, and has the potential to initiate and enhance immune responses against tumors in cancer treatment. It is suggested that necroptosis, mediated by EVs, can control the migration, proliferation, and invasion of tumor cells [120]. In a recent study utilizing proteomic analysis on enriched EVs, the release of cytokines specific to cell death, along with the underlying processes regulated during apoptosis and necroptosis, was revealed [123]. Proteomic analysis of EVs during necroptosis showed an additional regulatory mechanism in the early stage of necroptosis. This mechanism is mediated by specific EV cargoes that reshape the tumor microenvironment, inducing both adaptive and innate immune responses [124].

### 2.6. Extracellular Vesicles in the Aging Process

The process of aging is a gradual, progressive, and inevitable phenomenon that affects all living organisms. It involves both genetic and environmental factors. As the human body ages, it undergoes various changes at the cellular and molecular level, including inflammation and dysfunction of macromolecules. This can lead to the development of age-related disorders such as cancer, cardiovascular disease, and neurodegenerative lesions. To combat these effects of aging, researchers are exploring various strategies and trying to develop anti-aging therapies [125,126]. Recent studies suggest that EVs isolated from different types of stem cells may be a promising tool for combating aging-related inflammation and dysfunction of macromolecules [126,127,128]. EVs can transport a variety of bioactive molecules, such as microRNAs and proteins, that have the potential to modulate various cellular processes and promote tissue regeneration [129]. For example, EVs derived from stem cells have been shown to reduce inflammation and promote tissue repair in various organs, such as the heart, liver, and kidney [130].

Nine common preliminary cellular features are listed in the aging process of different organisms. The main hallmarks are genomic instability, telomere attrition, epigenetic changes, loss of proteostasis, dysregulated nutrient sensing, mitochondrial dysfunction, stem cell exhaustion, altered intercellular communication, and cellular senescence. EVs are involved in regulating some of these processes (Table 1) [131,132].

Two types of cellular aging can be distinguished based on their underlying mechanisms. One of them is senescence-associated secretory phenotype (SASP), which is common in the elderly people due to the exhaustion of the division limit, and is caused by the shortening of telomeres, which is held during cell divisions. The second is stress-induced premature senescence (SIPS), which is responsible for pathological aging, and is associated with the exposure to external factors, oxidative stress, oncogenes, and during the interaction of the cell’s DNA with damaging substances [132]. During permanent disruption of homeostasis, senescent cells can accumulate. This causes tissue dysfunction and tumorigenesis [142]. EVs are involved in both types of cellular aging. EVs derived from senescent cells can transfer senescence-associated molecules, such as microRNAs and proteins, to neighboring cells thereby causing cellular senescence. Conversely, EVs derived from non-senescent cells can promote the clearance of senescent cells and tissue regeneration [132]. Currently, there is no marker that is highly specific for senescent cells. However, there are markers that are commonly applicable [143]. It is recommended to determinate various markers, such as senescence-associated beta-galactosidase (SA β-gal) activity, lipofuscin, cyclin-dependent kinase inhibitors, secreted factors, and context-specific factors like p16INK4A and p21Cip1. Additionally, new markers are constantly being discovered and validated to improve the accuracy of senescence detection [142]. Adult stem cells have been observed to have a senescent phenotype with age. Studies have shown that reductions in senescent cells lead to reduced inflammation, macromolecular dysfunction, and improved progenitor cell function. EVs released from senescent cells have been linked to diseases such as cardiovascular disease, diabetes, neurological disorders, and vascular aging. Senescing EVs have the capacity to influence multiple proteins and miRs, thereby exacerbating age-related diseases [125]. Several preclinical studies have suggested that EVs from stem cells, and other sources have potential therapeutic applications in age-related diseases [144]. For instance, EVs derived from mesenchymal stem cells have been demonstrated to promote tissue regeneration and reduce inflammation in various models of age-related diseases, including osteoarthritis, Alzheimer’s, and cardiovascular disease [145].

Research has shown that alpha-synuclein (α-Syn), a key protein involved in Parkinson’s disease pathology, can travel between neurons via exosomes. Astrocyte-derived exosomes also have been demonstrated to promote the formation of protein plaques in the brain [125,146]. EVs from patients with neurological disorders were found to contain genetic cargo altered in the form of miRNAs and tau proteins. Therefore, these molecules were utilized as disease markers [147].

Additionally, EVs from young donor cells have a rejuvenating effect on aged tissues, including the brain, heart, and muscle [148,149].

Furthermore, EVs are potential tool for “unwanted molecules” removal, such as beta-amyloid plaques, strongly associated with Alzheimer’s disease [146]. A study investigating the impact of EVs from bone marrow mesenchymal stromal cells (BMMSCs) on the development of multiple myeloma (MM) showed that EVs obtained from BMMSCs are associated with MM and stimulate the growth of tumors in vivo, whereas EVs obtained from healthy BMMSCs suppressed tumor growth [150]. Moreover, EVs derived from human serum have the capacity to encourage vascular remodeling and safeguard muscle damage in mice models suffering from acute hind limb ischemia. This suggests that EVs derived from human serum may have therapeutic potential in the treatment of ischemic cardiovascular diseases [151].

## 3. Future Development, Practical Applications, and Possible Limitations of the EVs-Therapy

Studies of EVs as potential therapeutic tools for difficult-to-treat disorders are rapidly growing. EVs are applicable in many medical and clinical sciences fields, such as dermatology and neurology. Due to their versatility and the possibility of the oncology mentioned in the above modification, they are often used as carriers of potentially therapeutic drugs, especially those that find it challenging to obtain a therapeutic concentration at the site of action, or to overcome various barriers in the human body. As the research was carried out, it began to be noticed that more benefits are brought not by multiple drugs and chemical compounds, but by structures such as RNA (mainly miRNA and siRNA), various inhibitors, and cytokines. These factors make it possible to obtain a long-lasting healing effect because they affect the symptoms of the disease and often treat the underlying cause [14].

EVs appear perfect candidates to deliver therapeutic drugs, presenting several advantages compared to liposomes or polymer-based techniques. Firstly, they usually exist in body fluids, and, consequently, they are stable in physiological conditions. Furthermore, they are less immunogenic and cytotoxic compared to polymerized vectors. Finally, EVs can transfer cargo to particular cells due to their membrane proteins and lipids that can be applied to specific receptors in the target cells. In particular, this aspect is essential to transport therapeutics to specific areas where polymeric vesicles or liposomes cannot reach, such as the brain. In addition, some studies indicate that exosomes may cross the BBB following active endocytosis mechanisms. Moreover, EVs can interact with the BBB, changing the barrier’s characteristics [152].

EVs exhibit extensive therapeutic potential thanks to their low immunogenicity and robust protein or gene transfer capacity. Recently, novel strategies have emerged to tailor exosomes according to specific needs. This involves genetically engineering cells to overexpress particular macromolecules, or modifying them to release exosomes with precise targeting molecules. One approach consists of culturing patient cells, genetically modifying them for targeted therapeutic protein/RNA delivery, isolating EVs from the cultures, and administering them to the patient. Another strategy entails genetically modified cells producing therapeutic EVs in vivo [153]. However, the clinical application of EVs as drug-delivery agents faces notable challenges. The first hurdle involves the low isolation yield and the presence of impurities, such as proteins or other EVs. Secondly, it is crucial to assess the impact of EVs in both physiological and pathological conditions to predict short- and long-term safety [154]. In anti-cancer therapies, distinguishing between tumor-cell- and healthy-cell-derived vesicles is essential. Innovative technologies have already been developed for this purpose, including microarrays, specific monoclonal antibodies, and RNA marker amplification strategies [155].

EVs-therapies are gaining popularity, especially in comparison to stem cell treatments, due to the notable advantages of EVs, such as lower immunogenicity and toxicity—crucial for autoimmune diseases [156]. These characteristics are determined by the source and content of EVs, influencing decisions on using auto- or allogeneic EVs in experimental therapies based on the disease and patient’s condition [157]. However, challenges arise during isolation, with the process impacting therapeutic properties. For instance, centrifugation, a crucial step, affects phosphatidylserine levels, potentially leading to blood clotting [158]. The release environment also affects EV quality, posing challenges in obtaining target microbubble amounts for experimental therapies [156]. Selecting the cells for EV derivation and the isolation process is critical, but challenges persist in choosing the administration technique. Intravenous injection exposes EVs to the immune system and phagocytosis, with potential organ accumulation, such as crossing the blood–brain barrier. Accumulation depends on EV quality and origin, sometimes causing therapeutic or side effects like local inflammation [159]. Intramuscular or subcutaneous administration is recommended to mitigate organ accumulation, as intravenous administration leads to deposits within 48 h, while the former extends this period to 14 days. A critical aspect of developing EV-based carriers is their stability in body fluids [154].

## 4. Conclusions

Extracellular vesicles play an important role in various cellular processes, contributing to the maintenance of homeostasis. Besides their pivotal and complex role in eliminating unwanted molecules, they also serve as essential mediators in cellular communication. Understanding the intricate connections between EVs and various cellular processes is crucial for creating a therapeutic strategy. This review delves into the diversity of EVs. It explores their potential therapeutic roles as significant mediators in physiological and pathological processes and sheds light on their potential for therapeutic interventions. The analysis also anticipates future challenges in translating these promising findings into clinical practice.

## Figures and Tables

**Table 1:** Application of EVs in the aging process.

Possible Application of EVs in the Aging Process	Characteristic	Reference
Genomic instability	EVs have been shown to transport DNA and RNA between cells, which can promote DNA damage repair and maintain genome stability.	[133]
Rejuvenation of mesenchymal stem cells	EVs derived from young mesenchymal stem cells (MSCs) were found to possess rejuvenating properties, reversing age-related changes in recipient aged MSCs by transferring bioactive molecules.	[134]
Regeneration	According to research, the utilization of EV-loaded hydrogels has demonstrated a promising approach to tissue repair and regeneration, exhibiting efficacy in tissue and organs like: skin, bones, cartilage, heart, nerves, the reproductive system, periodontal, hair, liver, and kidneys.	[135]
Anti-tumor regulation	EVs serve as gateways through which cancer cells release their genomic DNA (gDNA) into the extracellular space, which can then be taken up by circulating leukocytes, particularly neutrophils. This changes their functioning, making them more likely to form blood clots and develop inflammation.	[136]
Telomere attrition	EVs can induce telomere shortening and DNA damage in recipient cells, leading to aging and genomic instability. However, treatments that remove the RNA or protein content from EVs can reduce this effect. Additionally, EVs can also spread senescence-associated characteristics to nearby cells by inducing hypomethylation, thereby causing genomic instability.	[137]
Presentation of abnormal telomeres	The cell-free form of telomeric repeat-containing RNA (cfTERRA) is released in EVs. Research has shown that cancer patients have higher levels of cfTERRA in their blood plasma, and this rise is linked to telomere dysfunction and DNA damage in the parent cells.	[137]
Epigenetic changes	The transport of epigenetic regulators, including microRNAs and histones EVs, enables them to modulate gene expression and chromatin structure when transferred between cells.	[138]
Dysregulated nutrient sensing	Dysregulated nutrient sensing, a hallmark of aging, involves disrupted pathways related to insulin signaling and mTOR signaling. EVs play a role in this process by transporting hormones and growth factors involved in nutrient sensing, including insulin, and factors related to mTOR signaling. By influencing these pathways, EVs can contribute to the dysregulation of nutrient sensing observed in aging.	[139]
Mitochondrial dysfunction	The mitochondrial-lysosomal axis is a cellular system responsible for removing damaged components, particularly dysfunctional mitochondria. Disruption of this system, along with abnormal EV secretion, has been associated with the aging process and various diseases.	[113]
Stem cell exhaustion	EVs derived from aged cells can affect mesenchymal stem cells (MSCs) from bone marrow. When young MSCs are exposed to EVs from old MSCs, it activates the mTOR pathway, leads to increased expression of aging markers, and reduces pluripotency marker levels. Similarly, when bone marrow stem cells (BMSCs) are treated with EVs from aged bone marrow fluids, it induces stem cell senescence and impairs their ability to differentiate into bone cells. Additionally, EVs carrying miR-34a, generated by oxidative stress in muscle cells, can suppress a protein called SIRT1 and promote senescence and death in BMSCs.	[137,140]
Altered intercellular communication	EVs can transport various signaling molecules, including cytokines and growth factors, between cells. By carrying these signaling molecules, EVs facilitate intercellular communication and contribute to the regulation of tissue homeostasis.	[141]
Cellular senescence	Cellular senescence is the loss of replicative potential in a normally dividing cell. EVs can promote or suppress cellular senescence by transferring senescence-associated molecules, such as microRNAs and proteins. Senescent cells are also characterized by an increased amount of pro-inflammatory cytokines, chemokines, tissue-damaging proteases, and other factors that can alter stem and progenitor cell function, hemostatic factors, and growth factors.	[125]

## Data Availability

No new data were created or analyzed in this study. Data sharing is not applicable to this article.

## References

[1] Abels E.R., Breakefield X.O. (2016). Introduction to Extracellular Vesicles: Biogenesis, RNA Cargo Selection, Content, Release, and Uptake. Cell. Mol. Neurobiol..

[2] Kalra H., Drummen G.P.C., Mathivanan S. (2016). Focus on extracellular vesicles: Introducing the next small big thing. Int. J. Mol. Sci..

[3] Margolis L., Sadovsky Y. (2019). The biology of extracellular vesicles: The known unknowns. PLoS Biol..

[4] Turchinovich A., Drapkina O., Tonevitsky A. (2019). Transcriptome of extracellular vesicles: State-of-the-art. Front. Immunol..

[5] Krylova S.V., Feng D. (2023). The Machinery of Exosomes: Biogenesis, Release, and Uptake. Int. J. Mol. Sci..

[6] Gould S.J., Booth A.M., Hildreth J.E.K. (2003). The Trojan exosome hypothesis. Proc. Natl. Acad. Sci. USA.

[7] Zaborowski M.P., Balaj L., Breakefield X.O., Lai C.P. (2015). Extracellular Vesicles: Composition, Biological Relevance, and Methods of Study. Bioscience.

[8] Sedgwick A.E., D’Souza-Schorey C. (2018). The biology of extracellular microvesicles. Traffic.

[9] Sheta M., Taha E.A., Lu Y., Eguchi T. (2023). Extracellular Vesicles: New Classification and Tumor Immunosuppression. Biology.

[10] Doyle L.M., Wang M.Z. (2019). Overview of extracellular vesicles, their origin, composition, purpose, and methods for exosome isolation and analysis. Cells.

[11] Meldolesi J. (2018). Exosomes and Ectosomes in Intercellular Communication. Curr. Biol..

[12] Maas S.L.N., Breakefield X.O., Weaver A.M. (2017). Extracellular Vesicles: Unique Intercellular Delivery Vehicles. Trends Cell Biol..

[13] Battistelli M., Falcieri E. (2020). Apoptotic Bodies: Particular Extracellular Vesicles Involved in Intercellular Communication. Biology.

[14] Szwedowicz U., Łapińska Z., Gajewska-Naryniecka A., Choromańska A. (2022). Exosomes and Other Extracellular Vesicles with High Therapeutic Potential: Their Applications in Oncology, Neurology, and Dermatology. Molecules.

[15] Cable J., Witwer K.W., Coffey R.J., Milosavljevic A., von Lersner A.K., Jimenez L., Pucci F., Barr M.M., Dekker N., Barman B. (2023). Exosomes, microvesicles, and other extracellular vesicles—A Keystone Symposia report. Ann. N. Y. Acad. Sci..

[16] Meldolesi J. (2019). Extracellular vesicles, news about their role in immune cells: Physiology, pathology and diseases. Clin. Exp. Immunol..

[17] Yates A.G., Pink R.C., Erdbrügger U., Siljander P.R., Dellar E.R., Pantazi P., Akbar N., Cooke W.R., Vatish M., Dias-Neto E. (2022). In sickness and in health: The functional role of extracellular vesicles in physiology and pathology in vivo: Part I: Health and Normal Physiology: Part I: Health and Normal Physiology. J. Extracell. Vesicles.

[18] Latifkar A., Hur Y.H., Sanchez J.C., Cerione R.A., Antonyak M.A. (2019). New insights into extracellular vesicle biogenesis and function. J. Cell Sci..

[19] Akers J.C., Gonda D., Kim R., Carter B.S., Chen C.C. (2013). Biogenesis of extracellular vesicles (EV): Exosomes, microvesicles, retrovirus-like vesicles, and apoptotic bodies. J. Neurooncol..

[20] Ibrahim S.A., Khan Y.S. (2020). Histology, Extracellular Vesicles. StatPearls. http://www.ncbi.nlm.nih.gov/pubmed/32965927.

[21] Popov L.D. (2022). Mitochondrial-derived vesicles: Recent insights. J. Cell. Mol. Med..

[22] Liu Y.J., Wang C. (2023). A review of the regulatory mechanisms of extracellular vesicles-mediated intercellular communication. Cell Commun. Signal..

[23] Singh A., Kukreti R., Saso L., Kukreti S. (2019). Oxidative Stress: A Key Modulator in Neurodegenerative Diseases. Molecules.

[24] Hajam Y.A., Rani R., Ganie S.Y., Sheikh T.A., Javaid D., Qadri S.S., Pramodh S., Alsulimani A., Alkhanani F.M., Harakeh S. (2022). Oxidative Stress in Human Pathology and Aging: Molecular Mechanisms and Perspectives. Cells.

[25] Qi H., Wang Y., Fa S., Yuan C., Yang L. (2021). Extracellular Vesicles as Natural Delivery Carriers Regulate Oxidative Stress under Pathological Conditions. Front. Bioeng. Biotechnol..

[26] Forman H.J., Zhang H. (2021). Targeting oxidative stress in disease: Promise and limitations of antioxidant therapy. Nat. Rev. Drug Discov..

[27] Chen Y., Jungsuwadee P., Vore M., Butterfield D.A., St. Clair D.K. (2007). Collateral damage in cancer chemotherapy: Oxidative stress in nontargeted tissues. Mol. Interv..

[28] Yarana C., Carroll D., Chen J., Chaiswing L., Zhao Y., Noel T., Alstott M., Bae Y., Dressler E.V., Moscow J.A. (2018). Extracellular vesicles released by cardiomyocytes in a doxorubicin-induced cardiac injury mouse model contain protein biomarkers of early cardiac injury. Clin. Cancer Res..

[29] Connolly K.D., Rees D.A., James P.E. (2021). Role of adipocyte-derived extracellular vesicles in vascular inflammation. Free Radic. Biol. Med..

[30] Chiaradia E., Tancini B., Emiliani C., Delo F., Pellegrino R.M., Tognoloni A., Urbanelli L., Buratta S. (2021). Extracellular Vesicles under Oxidative Stress Conditions: Biological Properties and Physiological Roles. Cells.

[31] Yang W., Zou B., Hou Y., Yan W., Chen T., Qu S. (2019). Extracellular vesicles in vascular calcification. Clin. Chim. Acta.

[32] Akbar N., Paget D., Choudhury R.P. (2021). Extracellular Vesicles in Innate Immune Cell Programming. Biomedicines.

[33] Yarana C., St. Clair D.K. (2017). Chemotherapy-Induced Tissue Injury: An Insight into the Role of Extracellular Vesicles-Mediated Oxidative Stress Responses. Antioxidants.

[34] Bodega G., Alique M., Puebla L., Carracedo J., Ramírez R.M. (2019). Microvesicles: ROS scavengers and ROS producers. J. Extracell. Vesicles.

[35] Soleti R., Lauret E., Andriantsitohaina R., Martínez M.C. (2012). Internalization and induction of antioxidant messages by microvesicles contribute to the antiapoptotic effects on human endothelial cells. Free Radic. Biol. Med..

[36] Yao J., Zheng J., Cai J., Zeng K., Zhou C., Zhang J., Li S., Li H., Chen L., He L. (2019). Extracellular vesicles derived from human umbilical cord mesenchymal stem cells alleviate rat hepatic ischemia-reperfusion injury by suppressing oxidative stress and neutrophil inflammatory response. FASEB J..

[37] Bodart-Santos V., de Carvalho L.R.P., de Godoy M.A., Batista A.F., Saraiva L.M., Lima L.G., Abreu C.A., De Felice F.G., Galina A., Mendez-Otero R. (2019). Extracellular vesicles derived from human Wharton’s jelly mesenchymal stem cells protect hippocampal neurons from oxidative stress and synapse damage induced by amyloid-β oligomers. Stem Cell Res. Ther..

[38] Mahmoud A.M., Wilkinson F.L., McCarthy E.M., Moreno-Martinez D., Langford-Smith A., Romero M., Duarte J., Alexander M.Y. (2017). Endothelial microparticles prevent lipid-induced endothelial damage via Akt/eNOS signaling and reduced oxidative stress. FASEB J..

[39] Wang T., Jian Z., Baskys A., Yang J., Li J., Guo H., Hei Y., Xian P., He Z., Liet Z. (2020). MSC-derived exosomes protect against oxidative stress-induced skin injury via adaptive regulation of the NRF2 defense system. Biomaterials.

[40] Zhou Y., Xu H., Xu W., Wang B., Wu H., Tao Y., Zhang B., Wang M., Mao F., Yan Y. (2013). Exosomes released by human umbilical cord mesenchymal stem cells protect against cisplatin-induced renal oxidative stress and apoptosis in vivo and in vitro. Stem Cell Res. Ther..

[41] Extracellular Vesicles Derived from Human Placental Mesenchymal Stem Cells Alleviate Experimental Colitis in Mice by Inhibiting Inflammation and Oxidative Stress. https://www.spandidos-publications.com/10.3892/ijmm.2020.4679.

[42] Sun T., Ding Z.X., Luo X., Liu Q.S., Cheng Y. (2020). Blood Exosomes Have Neuroprotective Effects in a Mouse Model of Parkinson’s Disease. Oxid. Med. Cell. Longev..

[43] Dong P., Zhang Y., Yan D.Y., Wang Y., Xu X., Zhao Y.C., Xiao T.T. (2020). Protective Effects of Human Milk-Derived Exosomes on Intestinal Stem Cells Damaged by Oxidative Stress. Cell Transplant..

[44] Burger D., Turner M., Munkonda M.N., Touyz R.M. (2016). Endothelial Microparticle-Derived Reactive Oxygen Species: Role in Endothelial Signaling and Vascular Function. Oxid. Med. Cell. Longev..

[45] Zhang Q., Shang M., Zhang M., Wang Y., Chen Y., Wu Y., Liu M., Song J., Liu Y. (2016). Microvesicles derived from hypoxia/reoxygenation-treated human umbilical vein endothelial cells promote apoptosis and oxidative stress in H9c2 cardiomyocytes. BMC Cell Biol..

[46] Rastogi S., Hwang A., Chan J., Wang J.Y.J. (2018). Extracellular vesicles transfer nuclear Abl-dependent and radiation-induced miR-34c into unirradiated cells to cause bystander effects. Mol. Biol. Cell.

[47] Xi X.J., Zeng J.J., Lu Y., Chen S.H., Jiang Z.W., He P.J., Mi H. (2020). Extracellular vesicles enhance oxidative stress through P38/NF-kB pathway in ketamine-induced ulcerative cystitis. J. Cell Mol. Med..

[48] Janiszewski M., Do Carmo A.O., Pedro M.A., Silva E., Knobel E., Laurindo F.R.M. (2004). Platelet-derived exosomes of septic individuals possess proapoptotic NAD(P)H oxidase activity: A novel vascular redox pathway. Crit. Care Med..

[49] Mostefai H.A., Meziani F., Mastronardi M.L., Agouni A., Heymes C., Sargentini C., Asfar P., Martinez M.C., Andriantsitohaina R. (2012). Circulating Microparticles from Patients with Septic Shock Exert Protective Role in Vascular Function. Am. J. Respir. Crit. Care Med..

[50] Manèek-Keber M., Frank-Bertoncelj M., Hafner-Bratkovič I., Smole A., Zorko M., Pirher N., Hayer S., Kralj-Iglič V., Rozman B., Ilc N. (2015). Toll-like receptor 4 senses oxidative stress mediated by the oxidation of phospholipids in extracellular vesicles. Sci. Signal.

[51] Becker A., Thakur B.K., Weiss J.M., Kim H.S., Peinado H., Lyden D. (2016). Extracellular Vesicles in Cancer: Cell-to-Cell Mediators of Metastasis. Cancer Cell.

[52] Muralidharan-Chari V., Kohan H.G., Asimakopoulos A.G., Sudha T., Sell S., Kannan K., Boroujerdi M., Davis P.J., Mousa S.A. (2016). Microvesicle removal of anticancer drugs contributes to drug resistance in human pancreatic cancer cells. Oncotarget.

[53] Yu X., Harris S.L., Levine A.J. (2006). The regulation of exosome secretion: A novel function of the p53 protein. Cancer Res..

[54] Lespagnol A., Duflaut D., Beekman C., Blanc L., Fiucci G., Marine J.C., Vidal M., Amson R., Telerman A. (2008). Exosome secretion, including the DNA damage-induced p53-dependent secretory pathway, is severely compromised in TSAP6/Steap3-null mice. Cell Death Differ..

[55] Wallace K.B. (2007). Adriamycin-induced interference with cardiac mitochondrial calcium homeostasis. Cardiovasc. Toxicol..

[56] Heuvingh J., Bonneau S. (2009). Asymmetric oxidation of giant vesicles triggers curvature-associated shape transition and permeabilization. Biophys. J..

[57] Théry C., Ostrowski M., Segura E. (2009). Membrane vesicles as conveyors of immune responses. Nat. Rev. Immunol..

[58] Schwarz D.S., Blower M.D. (2016). The endoplasmic reticulum: Structure, function and response to cellular signaling. Cell Mol. Life Sci..

[59] Oakes S.A., Papa F.R. (2015). The role of endoplasmic reticulum stress in human pathology. Annu. Rev. Pathol..

[60] Ron D., Walter P. (2007). Signal integration in the endoplasmic reticulum unfolded protein response. Nat. Rev. Mol. Cell Biol..

[61] Schröder M., Kaufman R.J. (2005). The mammalian unfolded protein response. Annu. Rev. Biochem..

[62] Wu J., Rutkowski D.T., Dubois M., Swathirajan J., Saunders T., Wang J., Song B., Yau G.D., Kaufman R.J. (2007). ATF6alpha optimizes long-term endoplasmic reticulum function to protect cells from chronic stress. Dev. Cell.

[63] Zhang K., Kaufman R.J. (2008). From endoplasmic-reticulum stress to the inflammatory response. Nature.

[64] Almeida L.M., Pinho B.R., Duchen M.R., Oliveira J.M.A. (2022). The PERKs of mitochondria protection during stress: Insights for PERK modulation in neurodegenerative and metabolic diseases. Biol. Rev. Camb. Philos. Soc..

[65] Di Conza G., Ho P.C. (2020). ER Stress Responses: An Emerging Modulator for Innate Immunity. Cells.

[66] Furmanik M., van Gorp R., Whitehead M., Ahmad S., Bordoloi J., Kapustin A., Schurgers L.J., Shanahan C.M. (2021). Endoplasmic Reticulum Stress Mediates Vascular Smooth Muscle Cell Calcification via Increased Release of Grp78 (Glucose-Regulated Protein, 78 kDa)-Loaded Extracellular Vesicles. Arterioscler. Thromb. Vasc. Biol..

[67] Cianciaruso C., Phelps E.A., Pasquier M., Hamelin R., Demurtas D., Alibashe Ahmed M., Piemonti L., Hirosue S., Swartz M.A., De Palma M. (2017). Primary Human and Rat β-Cells Release the Intracellular Autoantigens GAD65, IA-2, and Proinsulin in Exosomes Together With Cytokine-Induced Enhancers of Immunity. Diabetes.

[68] Weiss C., Kornicka-Grabowska K., Mularczyk M., Siwinska N., Marycz K. (2020). Extracellular Microvesicles (MV’s) Isolated from 5-Azacytidine-and-Resveratrol-Treated Cells Improve Viability and Ameliorate Endoplasmic Reticulum Stress in Metabolic Syndrome Derived Mesenchymal Stem Cells. Stem Cell Rev. Rep..

[69] Wang Z., Jiao P., Zhong Y., Ji H., Zhang Y., Song H., Du H., Ding X., Wu H. (2022). The Endoplasmic Reticulum-Stressed Head and Neck Squamous Cell Carcinoma Cells Induced Exosomal miR-424-5p Inhibits Angiogenesis and Migration of Humanumbilical Vein Endothelial Cells Through LAMC1-Mediated Wnt/β-Catenin Signaling Pathway. Cell Transplant..

[70] Osman A., Benameur T., Korashy H.M., Zeidan A., Agouni A. (2020). Interplay between Endoplasmic Reticulum Stress and Large Extracellular Vesicles (Microparticles) in Endothelial Cell Dysfunction. Biomedicines.

[71] Agouni A., Osman A., El Gamal H., Pasha M. (2020). Endoplasmic Reticulum (ER) stress-generated microparticles self-perpetuate ER stress and mediate endothelial cell dysfunction independently of cell survival. FASEB J..

[72] Lu C., Shi W., Hu W., Zhao Y., Zhao X., Dong F., Xin Y., Peng T., Liu C. (2022). Endoplasmic reticulum stress promotes breast cancer cells to release exosomes circ_0001142 and induces M2 polarization of macrophages to regulate tumor progression. Pharmacol. Res..

[73] Kim T.W., Ko S.G. (2021). The Herbal Formula JI017 Induces ER Stress via Nox4 in Breast Cancer Cells. Antioxidants.

[74] Aydin Y., Koksal A.R., Reddy V., Lin D., Osman H., Heidari Z., Rhadhi S.M., Wimley W.C., Parsi M.A., Dash S. (2021). Extracellular Vesicle Release Promotes Viral Replication during Persistent HCV Infection. Cells.

[75] Ye J., Liu X. (2022). Interactions between endoplasmic reticulum stress and extracellular vesicles in multiple diseases. Front. Immunol..

[76] Melo S.A., Sugimoto H., O’Connell J.T., Kato N., Villanueva A., Vidal A., Qiu L., Vitkin E., Perelman L.T., Melo C.A. (2014). Cancer exosomes perform cell-independent microRNA biogenesis and promote tumorigenesis. Cancer Cell.

[77] Chen C., Luo F., Liu X., Lu L., Xu H., Yang Q., Xue J., Shi L., Li J., Zhang A. (2017). NF-kB-regulated exosomal miR-155 promotes the inflammation associated with arsenite carcinogenesis. Cancer Lett..

[78] Felicetti F., De Feo A., Coscia C., Puglisi R., Pedini F., Pasquini L., Bellenghi M., Errico M.C., Pagani E., Carè A. (2016). Exosome-mediated transfer of miR-222 is sufficient to increase tumor malignancy in melanoma. J. Transl. Med..

[79] Liu Y., Zhao L., Li D., Yin Y., Zhang C., Li J., Zhang Y. (2013). Microvesicle-delivery miR-150 promotes tumorigenesis by up-regulating VEGF, and the neutralization of miR-150 attenuate tumor development. Protein Cell.

[80] Wang M., Kaufman R.J. (2014). The impact of the endoplasmic reticulum protein-folding environment on cancer development. Nat. Rev. Cancer.

[81] Choi D., Lee T.H., Spinelli C., Chennakrishnaiah S., D’Asti E., Rak J. (2017). Extracellular vesicle communication pathways as regulatory targets of oncogenic transformation. Semin. Cell Dev. Biol..

[82] Wu C.H., Silvers C.R., Messing E.M., Lee Y.F. (2019). Bladder cancer extracellular vesicles drive tumorigenesis by inducing the unfolded protein response in endoplasmic reticulum of nonmalignant cells. J. Biol. Chem..

[83] Jia L.X., Zhang W.M., Li T.T., Liu Y., Piao C.M., Ma Y.C., Lu Y., Wang Y., Liu T.T., Qi Y.F. (2017). ER stress dependent microparticles derived from smooth muscle cells promote endothelial dysfunction during thoracic aortic aneurysm and dissection. Clin. Sci..

[84] Safiedeen Z., Rodríguez-Gómez I., Vergori L., Soleti R., Vaithilingam D., Douma I., Agouni A., Leiber D., Dubois S., Simard G. (2017). Temporal Cross Talk Between Endoplasmic Reticulum and Mitochondria Regulates Oxidative Stress and Mediates Microparticle-Induced Endothelial Dysfunction. Antioxid. Redox Signal.

[85] Shanahan C.M. (2013). Mechanisms of vascular calcification in CKD-evidence for premature ageing?. Nat. Rev. Nephrol..

[86] Karwowski W., Naumnik B., Szczepański M., Myśliwiec M. (2012). The mechanism of vascular calcification—A systematic review. Med. Sci. Monit..

[87] Sage A.P., Tintut Y., Demer L.L. (2010). Regulatory mechanisms in vascular calcification. Nat. Rev. Cardiol..

[88] Qin Y., Wang Y., Liu O., Jia L., Fang W., Du J., Wei Y. (2017). Tauroursodeoxycholic Acid Attenuates Angiotensin II Induced Abdominal Aortic Aneurysm Formation in Apolipoprotein E-deficient Mice by Inhibiting Endoplasmic Reticulum Stress. Eur. J. Vasc. Endovasc. Surg..

[89] Greening D.W., Gopal S.K., Xu R., Simpson R.J., Chen W. (2015). Exosomes and their roles in immune regulation and cancer. Semin. Cell Dev. Biol..

[90] Youle R.J., Narendra D.P. (2011). Mechanisms of mitophagy. Nat. Rev. Mol. Cell Biol..

[91] Baixauli F., López-Otín C., Mittelbrunn M. (2014). Exosomes and Autophagy: Coordinated Mechanisms for the Maintenance of Cellular Fitness. Front. Immunol..

[92] Hetz C., Zhang K., Kaufman R.J. (2020). Mechanisms, regulation and functions of the unfolded protein response. Nat. Rev. Mol. Cell Biol..

[93] Mizushima N., Levine B., Cuervo A.M., Klionsky D.J. (2008). Autophagy fights disease through cellular self-digestion. Nature.

[94] Arias E., Cuervo A.M. (2011). Chaperone-mediated autophagy in protein quality control. Curr. Opin. Cell Biol..

[95] Xing H., Tan J., Miao Y., Lv Y., Zhang Q. (2021). Crosstalk between exosomes and autophagy: A review of molecular mechanisms and therapies. J. Cell. Mol. Med..

[96] Galluzzi L., Pietrocola F., Bravo-San Pedro J.M., Amaravadi R.K., Baehrecke E.H., Cecconi F., Codogno P., Debnath J., Gewirtz D.A., Karantza V. (2015). Autophagy in malignant transformation and cancer progression. EMBO J..

[97] Humeau J., Leduc M., Cerrato G., Loos F., Kepp O., Kroemer G. (2020). Phosphorylation of eukaryotic initiation factor-2α (eIF2α) in autophagy. Cell Death Dis..

[98] Kroemer G., Mariño G., Levine B. (2010). Autophagy and the integrated stress response. Mol. Cell.

[99] Roger A.J., Muñoz-Gómez S.A., Kamikawa R. (2017). The Origin and Diversification of Mitochondria. Curr. Biol..

[100] Todkar K., Chikhi L., Desjardins V., El-Mortada F., Pépin G., Germain M. (2021). Selective packaging of mitochondrial proteins into extracellular vesicles prevents the release of mitochondrial DAMPs. Nat. Commun..

[101] Nunnari J., Suomalainen A. (2012). Mitochondria: In sickness and in health. Cell.

[102] Picca A., Guerra F., Calvani R., Coelho-Júnior H.J., Landi F., Bucci C., Marzetti E. (2023). Mitochondrial-Derived Vesicles: The Good, the Bad, and the Ugly. Int. J. Mol. Sci..

[103] Zecchini V., Paupe V., Herranz-Montoya I., Janssen J., Wortel I.M.N., Morris J.L., Ferguson A., Chowdury S.R., Segarra-Mondejar M., Costa A.S.H. (2023). Fumarate induces vesicular release of mtDNA to drive innate immunity. Nature.

[104] Terešak P., Lapao A., Subic N., Boya P., Elazar Z., Simonsen A. (2022). Regulation of PRKN-independent mitophagy. Autophagy.

[105] Faas M.M., de Vos P. (2020). Mitochondrial function in immune cells in health and disease. Biochim. Biophys. Acta Mol. Basis Dis..

[106] Deus C.M., Tavares H., Beatriz M., Mota S., Lopes C. (2022). Mitochondrial Damage-Associated Molecular Patterns Content in Extracellular Vesicles Promotes Early Inflammation in Neurodegenerative Disorders. Cells.

[107] Marchi S., Guilbaud E., Tait S.W.G., Yamazaki T., Galluzzi L. (2023). Mitochondrial control of inflammation. Nat. Rev. Immunol..

[108] Green D.R. (2022). The Mitochondrial Pathway of Apoptosis: Part I: MOMP and Beyond. Cold Spring Harb. Perspect. Biol..

[109] Vringer E., Tait S.W.G. (2023). Mitochondria and cell death-associated inflammation. Cell Death Differ..

[110] De Gaetano A., Solodka K., Zanini G., Selleri V., Mattioli A.V., Nasi M., Pinti M. (2021). Molecular Mechanisms of mtDNA-Mediated Inflammation. Cells.

[111] Oka T., Hikoso S., Yamaguchi O., Taneike M., Takeda T., Tamai T., Oyabu J., Murakawa T., Nakayama H., Nishida K. (2012). Mitochondrial DNA that escapes from autophagy causes inflammation and heart failure. Nature.

[112] Banoth B., Cassel S.L. (2018). Mitochondria in innate immune signaling. Transl. Res..

[113] Picca A., Guerra F., Calvani R., Bucci C., Lo Monaco M.R., Bentivoglio A.R., Coelho-Júnior H.J., Landi F., Bernabei R., Marzetti E. (2019). Mitochondrial Dysfunction and Aging: Insights from the Analysis of Extracellular Vesicles. Int. J. Mol. Sci..

[114] Picca A., Calvani R., Coelho-junior H.J., Marzetti E. (2021). Cell Death and Inflammation: The Role of Mitochondria in Health and Disease. Cells.

[115] Di Mambro T., Pellielo G., Agyapong E.D., Carinci M., Chianese D., Giorgi C., Morciano G., Patergnani S., Pinton P., Rimessi A. (2023). The Tricky Connection between Extracellular Vesicles and Mitochondria in Inflammatory-Related Diseases. Int. J. Mol. Sci..

[116] Zhao M., Wang Y., Li L., Liu S., Wang C., Yuan Y., Yang G., Chen Y., Cheng J., Lu Y. (2021). Mitochondrial ROS promote mitochondrial dysfunction and inflammation in ischemic acute kidney injury by disrupting TFAM-mediated mtDNA maintenance. Theranostics.

[117] Quan Y., Xin Y., Tian G., Zhou J., Liu X. (2020). Mitochondrial ROS-Modulated mtDNA: A Potential Target for Cardiac Aging. Oxid. Med. Cell. Longev..

[118] Hadian K., Stockwell B.R. (2023). The therapeutic potential of targeting regulated non-apoptotic cell death. Nat. Rev. Drug Discov..

[119] Teng Y., Xu D., Yang X., Tang H., Tao X., Fan Y., Ding Y. (2023). The Emerging Roles of Pyroptosis, Necroptosis, and Ferroptosis in Non-Malignant Dermatoses: A Review. J. Inflamm. Res..

[120] Gong Y., Fan Z., Luo G., Yang C., Huang Q., Fan K., Cheng H., Jin K., Ni Q., Yu X. (2019). The role of necroptosis in cancer biology and therapy. Mol. Cancer.

[121] Yang Y.C., Jiang Q., Yang K.P., Wang L., Sethi G., Ma Z. (2024). Extracellular vesicle-mediated ferroptosis, pyroptosis, and necroptosis: Potential clinical applications in cancer therapy. Cell Death Discov..

[122] Li K., Qiu J., Pan J., Pan J.P. (2022). Pyroptosis and Its Role in Cervical Cancer. Cancers.

[123] Tanzer M.C., Frauenstein A., Stafford C.A., Phulphagar K., Mann M., Meissner F. (2020). Quantitative and Dynamic Catalogs of Proteins Released during Apoptotic and Necroptotic Cell Death. Cell Rep..

[124] Shlomovitz I., Erlich Z., Arad G., Edry-Botzer L., Zargarian S., Cohen H., Manko T., Ofir-Birin Y., Cooks T., Regev-Rudzki N. (2021). Proteomic analysis of necroptotic extracellular vesicles. Cell Death Dis..

[125] Saheera S., Potnuri A.G., Krishnamurthy P. (2020). Nano-Vesicle (Mis)Communication in Senescence-Related Pathologies. Cells.

[126] Das M., Kale V. (2021). Involvement of extracellular vesicles in aging process and their beneficial effects in alleviating aging-associated symptoms. Cell Biol. Int..

[127] Andjus P., Kosanović M., Milićević K., Gautam M., Vainio S.J., Jagečić D., Kozlova E.N., Pivoriūnas A., Chachques J.C., Sakaj M. (2020). Extracellular Vesicles as Innovative Tool for Diagnosis, Regeneration and Protection against Neurological Damage. Int. J. Mol. Sci..

[128] Manni G., Buratta S., Pallotta M.T., Chiasserini D., Di Michele A., Emiliani C., Giovagnoli S., Pascucci L., Romani R., Bellezza I. (2023). Extracellular Vesicles in Aging: An Emerging Hallmark?. Cells.

[129] Pinson M.R., Chung D.D., Adams A.M., Scopice C., Payne E.A., Sivakumar M., Miranda R.C. (2021). Extracellular Vesicles in Premature Aging and Diseases in Adulthood Due to Developmental Exposures. Aging Dis..

[130] Zhang B., Tian X., Hao J., Xu G., Zhang W. (2020). Mesenchymal Stem Cell-Derived Extracellular Vesicles in Tissue Regeneration. Cell Transplant..

[131] López-Otín C., Blasco M.A., Partridge L., Serrano M., Kroemer G. (2013). The Hallmarks of Aging. Cell.

[132] Mas-Bargues C., Alique M. (2023). Extracellular Vesicles as ‘Very Important Particles’ (VIPs) in Aging. Int. J. Mol. Sci..

[133] Elzanowska J., Semira C., Costa-Silva B. (2021). DNA in extracellular vesicles: Biological and clinical aspects. Mol. Oncol..

[134] Khanh V.C., Yamashita T., Ohneda K., Tokunaga C., Kato H., Osaka M., Hiramatsu Y., Ohneda O. (2020). Rejuvenation of mesenchymal stem cells by extracellular vesicles inhibits the elevation of reactive oxygen species. Sci. Rep..

[135] Ju Y., Hu Y., Yang P., Xie X., Fang B. (2022). Extracellular vesicle-loaded hydrogels for tissue repair and regeneration. Mater. Today Bio.

[136] Chennakrishnaiah S., Meehan B., D’Asti E., Montermini L., Lee T.H., Karatzas N., Buchanan M., Tawil N., Choi D., Divangahi M. (2018). Leukocytes as a reservoir of circulating oncogenic DNA and regulatory targets of tumor-derived extracellular vesicles. J. Thromb. Haemost..

[137] Yin Y., Chen H., Wang Y., Zhang L., Wang X. (2021). Roles of extracellular vesicles in the aging microenvironment and age-related diseases. J. Extracell. Vesicles.

[138] Moutinho C., Esteller M. (2017). MicroRNAs and Epigenetics. Adv. Cancer Res..

[139] Guenther G.G., Peralta E.R., Rosales K.R., Wong S.Y., Siskind L.J., Edinger A.L. (2008). Ceramide starves cells to death by downregulating nutrient transporter proteins. Proc. Natl. Acad. Sci. USA.

[140] Fulzele S., Mendhe B., Khayrullin A., Johnson M., Kaiser H., Liu Y., Isales C.M., Hamrick M.W. (2019). Muscle-derived miR-34a increases with age in circulating extracellular vesicles and induces senescence of bone marrow stem cells. Aging (Albany NY).

[141] Colombo M., Raposo G., Théry C. (2014). Biogenesis, secretion, and intercellular interactions of exosomes and other extracellular vesicles. Annu. Rev. Cell Dev. Biol..

[142] Mcilvenna L.C., Whitham M. (2022). Exercise, healthy ageing, and the potential role of small extracellular vesicles. J. Physiol..

[143] Gorgoulis V., Adams P.D., Alimonti A., Bennett D.C., Bischof O., Bishop C., Campisi J., Collado M., Evangelou K., Ferbeyre G. (2019). Cellular Senescence: Defining a Path Forward. Cell.

[144] Ren K. (2019). Exosomes in perspective: A potential surrogate for stem cell therapy. Odontology.

[145] Ryan S.T., Ryan S.T., Hosseini-Beheshti E., Afrose D., Ding X., Xia B., Grau G., Little C., McClements L., Li J. (2021). Extracellular Vesicles from Mesenchymal Stromal Cells for the Treatment of Inflammation-Related Conditions. Int. J. Mol. Sci..

[146] Vidal M. (2019). Exosomes: Revisiting their role as ‘garbage bags’. Traffic.

[147] Fowler C.D., Hill A.F. (2019). Extracellular Vesicles and Neurodegenerative Diseases. J. Neurosci..

[148] Franceschi C., Garagnani P., Morsiani C., Conte M., Santoro A., Grignolio A., Monti D., Capri M., Salvioli S. (2018). The continuum of aging and age-related diseases: Common mechanisms but different rates. Front. Med..

[149] Wang W., Wang L., Ruan L., Oh J., Dong X., Zhuge Q., Su D.M. (2018). Extracellular vesicles extracted from young donor serum attenuate inflammaging via partially rejuvenating aged T-cell immunotolerance. FASEB J..

[150] Roccaro A.M., Sacco A., Maiso P., Azab A.K., Tai Y.T., Reagan M., Azab F., Flores L.M., Campigotto F., Weller E. (2013). BM mesenchymal stromal cell–derived exosomes facilitate multiple myeloma progression. J. Clin. Investig..

[151] Cavallari C., Ranghino A., Tapparo M., Cedrino M., Figliolini F., Grange C., Giannachi V., Garneri P., Deregibus M.C., Collino F. (2017). Serum-derived extracellular vesicles (EVs) impact on vascular remodeling and prevent muscle damage in acute hind limb ischemia. Sci. Rep..

[152] Daltro S.R.T., Meira C.S., Santos I.P., Ribeiro dos Santos R., Soares M.B.P. (2020). Mesenchymal Stem Cells and Atopic Dermatitis: A Review. Front. Cell Dev. Biol..

[153] Leung D.Y.M. (2000). Atopic dermatitis: New insights and opportunities for therapeutic intervention. J. Allergy Clin. Immunol..

[154] Shin K.O., Ha D.H., Kim J.O., Crumrine D.A., Meyer J.M., Wakefield J.S., Lee Y., Kim B., Kim S., Kim H.K. (2020). Exosomes from Human Adipose Tissue-Derived Mesenchymal Stem Cells Promote Epidermal Barrier Repair by Inducing de Novo Synthesis of Ceramides in Atopic Dermatitis. Cells.

[155] Cho B.S., Kim J.O., Ha D.H., Yi Y.W. (2018). Exosomes derived from human adipose tissue-derived mesenchymal stem cells alleviate atopic dermatitis. Stem Cell Res. Ther..

[156] Cai Y., Liu W., Lian L., Xu Y., Bai X., Xu S., Zhang J. (2020). Stroke treatment: Is exosome therapy superior to stem cell therapy?. Biochimie.

[157] Mendt M., Rezvani K., Shpall E. (2019). Mesenchymal stem cell-derived exosomes for clinical use. Bone Marrow Transplant..

[158] Van der Pol E., Böing A.N., Harrison P., Sturk A., Nieuwland R. (2012). Classification, functions, and clinical relevance of extracellular vesicles. Pharmacol. Rev..

[159] Van Hezel M.E., Nieuwland R., van Bruggen R., Juffermans N.P. (2017). The Ability of Extracellular Vesicles to Induce a Pro-Inflammatory Host Response. Int. J. Mol. Sci..

